# Levamisole Impairs Vascular Function by Blocking α-Adrenergic Receptors and Reducing NO Bioavailability in Rabbit Renal Artery

**DOI:** 10.1007/s12012-024-09879-w

**Published:** 2024-06-14

**Authors:** Sol Guerra-Ojeda, Patricia Marchio, Andrea Suarez, Martin Aldasoro, Soraya L. Valles, Patricia Genoves, Jose M. Vila, Maria D. Mauricio

**Affiliations:** 1 Department of Physiology, School of Medicine, University of Valencia, Blasco Ibañez, 15, 46010 Valencia, Spain; 2 INCLIVA, Institute of Health Research, Valencia, Spain; 3 Center for Biomedical Research Network on Cardiovascular Diseases (CIBER-CV), Madrid, Spain

**Keywords:** Levamisole, Renovascular tone, Endothelial dysfunction, Oxidative stress

## Abstract

Levamisole is an anthelmintic drug restricted to veterinary use but is currently detected as the most widely used cocaine cutting agent in European countries. Levamisole-adulterated cocaine has been linked to acute kidney injury, marked by a decrease in glomerular filtration rate, which involves reduced renal blood flow, but data on the alteration of renovascular response produced by levamisole are scarce. Renal arteries were isolated from healthy rabbits and used for isometric tension recording in organ baths and protein analysis. We provide evidence that depending on its concentration, levamisole modulates renovascular tone by acting as a non-selective α-adrenergic receptor blocker and down-regulates α_1_-adrenoceptor expression. Furthermore, levamisole impairs the endothelium-dependent relaxation induced by acetylcholine without modifying endothelial nitric oxide synthase (eNOS) expression. However, exposure to superoxide dismutase (SOD) partially prevents the impairment of ACh-induced relaxation by levamisole. This response is consistent with a down-regulation of SOD1 and an up-regulation of NADPH oxidase 4 (Nox4), suggesting that endothelial NO loss is due to increased local oxidative stress. Our findings demonstrate that levamisole can interfere with renal blood flow and the coordinated response to a vasodilator stimulus, which could worsen the deleterious consequences of cocaine use.

**Graphical Abstract:**

*EFS* electric field stimulation, *NA* noradrenaline, *AR* adrenergic receptor, *IP*_*3*_ inositol 1, 4, 5-trisphosphate, *cAMP* cyclic adenosine monophosphate, *mAChR* muscarinic acetylcholine receptor, *eNOS* endothelial nitric oxide synthase, *sGC* soluble guanylyl cyclase, *SOD* superoxide dismutase, *NOX4* NAPH oxidase 4

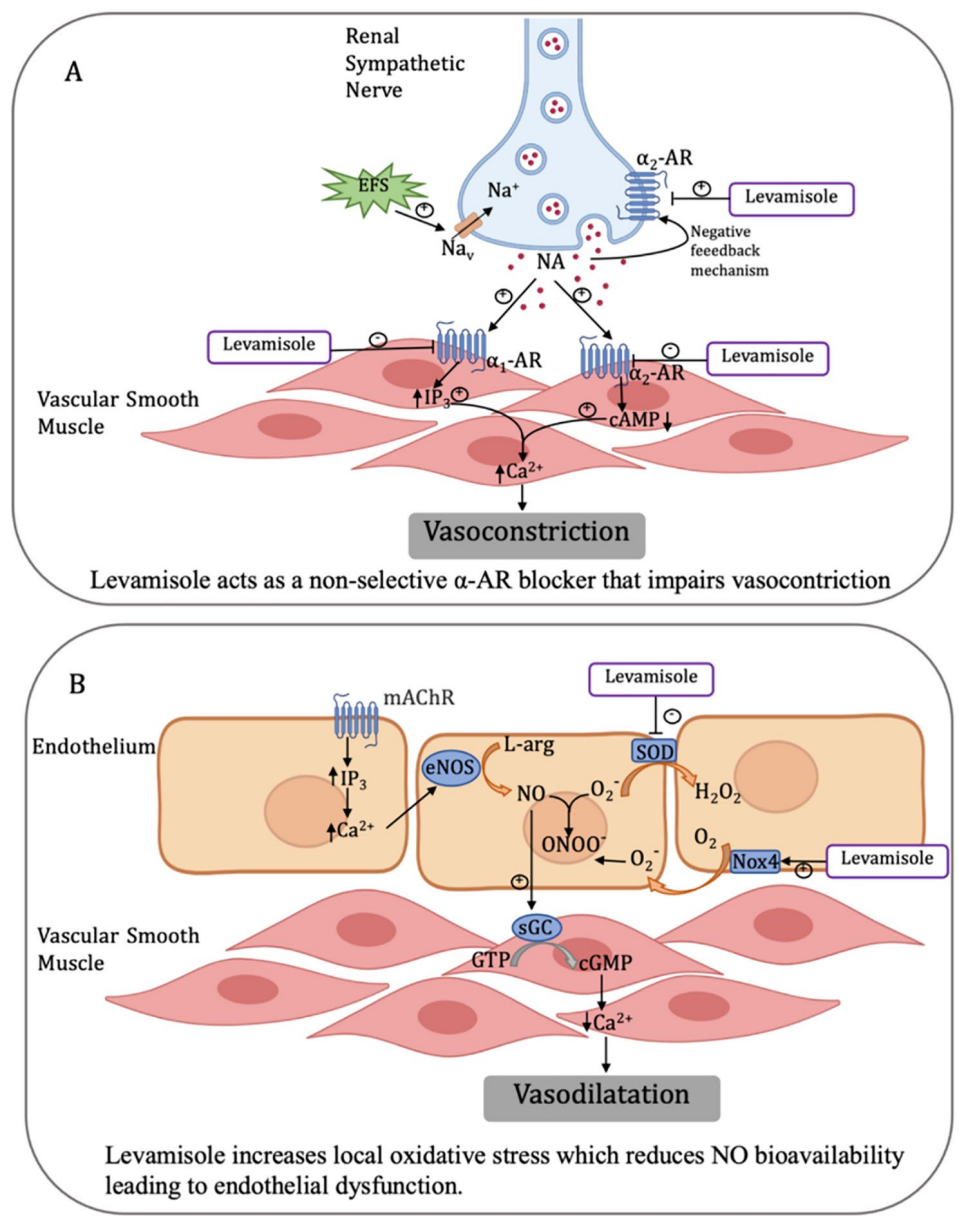

## Introduction

Levamisole is an imidazothiol-derived substance approved as an anthelmintic for veterinary use. It was previously used as an immunomodulator and chemotherapy adjuvant in humans. Nevertheless, it was withdrawn from the market in the US and most European countries for its adverse effects, such as agranulocytosis, leukoencephalopathy, and vasculitis [1–3]. Levamisole received an orphan drug designation from the European Medical Agency (EMA) in 2005 for its potential benefit for the treatment of nephrotic syndrome based on the results in experimental models [4]. However, the application for marketing authorization was withdrawn by the manufacturer in 2017 after the Committee for Medicinal Products for Human Use (CHMP) manifested methodological concerns about the main study carried out in children [5]. Since 2004, levamisole has been detected as the most widely used cocaine cutting agent in European countries [6–8]. It has been suggested that cocaine adulteration with levamisole is related to its cheapness [9], its physicochemical properties [10], and its potentiating effect on cocaine. In fact, it has been shown that levamisole enhances the action of cocaine in vivo in planaria and rats [11, 12], which is attributed to its combined action on monoamine oxidase blockade and nicotinic receptor activation, thus increasing dopaminergic transmission. Levamisole also acutely blocks noradrenaline (NA) reuptake but with a much lower potency than cocaine [13]. Its active metabolite is aminorex, an amphetamine-like substance [13], so it could be assumed that levamisole is also used to extend the effects of cocaine.

Levamisole-adulterated cocaine is associated with neutropenia and agranulocytosis, vasculitis, retiform purpura and other forms of skin necrosis. However, besides cardiac and cerebral complications, it has also been related to renal complications such as acute kidney injury [14–17], nephrotic syndrome [15, 16, 18, 19] and chronic kidney disease [14, 20].

The hallmark of acute kidney injury is a reduction in glomerular filtration rate, which implies an underlying alteration of haemodynamic regulation, usually defined by a sustained increase in renovascular resistance in response to decreased renal perfusion pressure, further compromising blood flow [21, 22]. This response can be attributed to several factors, such as an imbalance between the release of vasoconstrictors and vasodilators, especially nitric oxide (NO), oxidative stress and O_2_ supply [23], which ultimately leads to endothelial dysfunction. In this regard, levamisole has been shown to produce apoptosis and reduce eNOS and endothelin-1 expression in HUVECs-human umbilical vein endothelial cells [24]. Apoptosis appears to be related to oxidative stress as it is counteracted by antioxidants such as glutathione and N-acetylcysteine [24]. Collectively, these findings suggest that levamisole may exert a significant effect on the endothelium, contributing to the detrimental effect on the cardiovascular system. Nevertheless, the possibility that levamisole may lead to altered renovascular response has not been seriously considered. Against that background, the aim of the present study was to test the following hypotheses in rabbit renal arteries: (1) levamisole increases adrenergic contractile response, (2) levamisole causes endothelial dysfunction by reducing NO bioavailability or affecting endothelium-derived hyperpolarization (EDH), and (3) loss of endothelial NO is related to an increase in the vascular oxidative stress.

## Materials and Methods

### Animal Procedures

16-Week-old male New Zealand white rabbits (San Bernardo S.L., Spain) weighing 3 to 3.5 kg were euthanized with sodium thiopental (60 mg/kg i.v.) and, after laparotomy, renal arteries were removed and placed in chilled (4 °C) Krebs–Henseleit solution containing (in mM): NaCl, 115; KCl, 4.6; MgCl_2_·6H_2_O, 1.2; CaCl_2_, 2.5; NaHCO_3_, 25; glucose, 11.1 and disodium EDTA, 0.01 (pH 7.30–7.40 at 37 °C).

### Preparation of Renal Arteries for Vascular Reactivity

Dissected renal arteries were cleaned and cut into 3 mm-rings. Each ring was mounted in an organ bath containing 4 ml of Krebs–Henseleit with two L-shaped stainless-steel wires, as previously described [25]. The solution was gassed with 95% O_2_ and 5% CO_2_ at 37 °C. Changes in isometric force generation were recorded at 100 Hz using a PowerLab/8e data acquisition system and LabChart software v7.2.5 (ADInstruments, New Zealand). Arteries were allowed to equilibrate for 2 h to a resting tension of 1 g. The contractile capacity of vascular smooth muscle was then evaluated by the maximal response to KCl (60 mM). Functional endothelium was assessed by pre-constriction to NA (10^−7^ to 3 × 10^−7^ M, ~ 75% of the maximal contraction to KCl 60 mM) followed by endothelium-dependent relaxation to acetylcholine (ACh, 10^−6^ M). Only arteries relaxing > 70% were used for experiments. Then, cumulative response to levamisole (10^−6^ to 10^−3^ M) was assessed in renal rings precontracted with NA (10^−7^- to 3 × 10^−7^ M). The concentrations of levamisole used in the study were in the range of 10^−6^ M to 10^−3^ M, which is similar to the levels found in plasma samples collected in vivo (peak concentration range 50–112 µg/l) [26–28] and post-mortem (peak concentration 1106 µg/l) [28] from cocaine users. In another set of experiments, relaxing response to levamisole was evaluated in the absence and presence of endothelium-derived factor inhibitors such as N^G^-nitro-l-arginine methyl ester (L-NAME, 10^−4^ M), indomethacin (Indo, 10^−5^ M) and tetraethylammonium (TEA, 10^−3^ M), and adrenoceptors (AR) blockers such as prazosin (10^−6^ M), yohimbine (10^−6^ M), and propranolol (10^−6^ M). In the case of prazosin, NA concentration (10^−6^ to 3 × 10^−6^ M) was adjusted to achieve the same degree of precontraction between comparison groups. To further determine the effects of levamisole on vascular α_1_-AR, curves to cumulative phenylephrine (PE, 10^−9^ to 3 × 10^−5^ M) were performed in the absence and presence of levamisole (10^−5^ to 10^−3^ M).

To examine endothelium-dependent relaxation–response, curves to cumulative ACh (10^−9^ to 3 × 10^−6^ M) were performed in rings precontracted with NA (10^−6^ to 3 × 10^−6^ M) in the absence and presence of levamisole (10^−5^ to 10^−3^ M). Levamisole-induced oxidative stress was tested in curves to cumulative ACh in the absence and presence of SOD (200 U/ml) and levamisole (10^−3^ M). Endothelium-independent relaxation was assessed by cumulative addition of sodium nitroprusside (10^−9^ to 3 × 10^−6^ M) in renal rings precontracted with NA (10^−6^ to 3 × 10^−6^ M) in the absence and presence of levamisole (10^−3^ M). For acetylcholine and sodium nitroprusside curves incubated with levamisole, the NA concentration for each vascular ring was adjusted to achieve the same degree of precontraction between comparison groups.

### Measurement of Neurogenic Tone by Electrical Field Stimulation

For electric field stimulation (EFS) of sympathetic nerves to induce neurogenic tone, arteries were prepared as previously described [29]. EFS was applied by a Grass S88 stimulator (Grass Instruments) via two platinum electrodes positioned on each side and parallel to the axis of the arterial ring. Single square wave pulses (0.25 ms pulse duration, 8 Hz, at a supramaximal voltage of 20 V) were used. The train duration was 30 s with a stimulation interval of 5 min. In these conditions, EFS was applied to the renal rings before and after the addition of levamisole (10^−6^ to 10^−3^ M).

### Western Blot Analysis

Renal arteries were incubated in the organ bath in control conditions and the presence of levamisole (10^−5^ to 10^−3^ M) for 30 min at 37 °C. Then, arteries were snap-frozen in liquid nitrogen and homogenized in lysis buffer (0.125 m Tris–HCl, pH 6.8, 2% SDS, 19% glycerol and 1% v/v protease inhibitors). Tissue lysates were centrifuged at 12,000 rpm for 20 min at 4 °C. Protein concentrations were determined using bicinchoninic acid protein assay (Thermo Scientific, USA). Proteins (30 μg) were separated on SDS‐PAGE gels and transferred to polyvinylidene fluoride membranes. Subsequently, the membranes were blocked for 1 h at room temperature in blocking buffer (5% bovine serum albumin, 0.1% Tween 20 in Tris-buffered solution), followed by incubation overnight at 4 °C with primary antibody against either α_1A_-adrenergic (NOVUS, #NB100 78585), α_2A_-adrenergic (NOVUS, #NBP2-22452), eNOS (Signalway, #21170-2), Cu/Zn–SOD (SOD1, Thermo Fisher, # MA1-105), Mn–SOD (SOD2, Thermo Fisher, #PA5-30604), Nox4 (Invitrogen, #PA5-72922), α‐tubulin (Santa Cruz Biotechnology, #sc 5286) and β‐actin (NOVUS, #NB-600-501SS). After incubation with corresponding secondary antibodies for 1 h at room temperature, bands were detected by chemiluminescence method (Amersham Biosciences) and visualized by the digital image system ImageQuant LAS 4000 (GE Healthcare). Protein expression was quantified densitometrically with ImageJ (National Institutes of Health, Bethesda, MD, USA). α‐Tubulin or β‐actin were used as protein loading controls.

### Drugs and Solutions

Drugs were provided by Sigma Aldrich unless otherwise specified. Potassium chloride was obtained from Panreac. Indomethacin was dissolved in 100% ethanol. All other solutions were dissolved in distilled water. Stock solutions for organ baths were prepared daily in saline solution and kept on ice throughout the experiment. Levamisole and inhibitors were incubated for 30 min before measurements, except for SOD which was incubated for 45 min.

### Statistical Analysis

Values were summarized as mean ± SEM of *n* arteries (one for each animal). Relaxations were expressed as a percentage of inhibition of agonist-induced response and contractions as a percentage relative to the value of the maximum contraction at KCl (60 mM). pEC_50_ values (negative log of concentration required to elicit 50% of the maximum effect) were determined from individual concentration–response curves by nonlinear regression analysis. The statistical analysis was made in Prism 8.0.2 (GraphPad Software, Inc., San Diego, CA, USA) using Student’s *t*-test, one-way analysis of variance (ANOVA), or two-way ANOVA with Bonferroni’s post-test as appropriate, where *P* < 0.05 was considered significant.

## Results

### Levamisole Induces Relaxation by Blocking α-Adrenergic Receptors

**Fig. 1:**
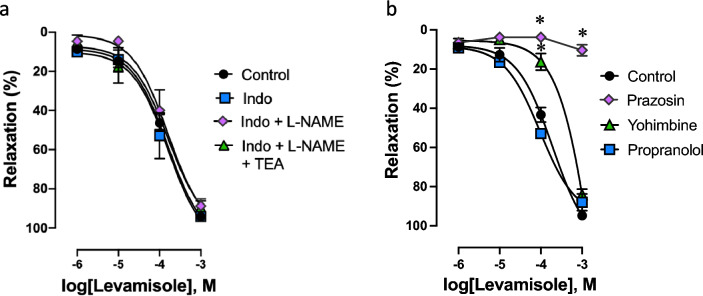
Levamisole induces relaxation in rabbit renal arteries precontracted with NA. **a** Relaxation–response curves to cumulative levamisole (10^−6^ to 10^−3^ M) in the absence (control) and presence of indomethacin (Indo, 10^−5^ M), a non-selective COX inhibitor, as well as consecutive additions of L-NAME (10^−4^ M), to block nitric oxide synthase and tetraethylammonium (TEA, 10^−3^ M), a calcium-activated K^+^ channels blocker. **b** Relaxation–response curves to cumulative levamisole in the absence (control) and presence of prazosin (10^−6^ M), to block α_1_-AR, yohimbine (10^−6^ M), to block α_2_-AR, and propranolol (10^−6^ M), to block β-AR. Data are means ± SEM (*n* = 3 to 6 arteries from different rabbits); *P < 0.05 vs. control

Relaxation of renal arteries was assessed after submaximal pre-constriction with NA (10^−7^ to 3 × 10^−7^ M). Levamisole produced concentration-dependent relaxation (*E*_max_ = 94.2 ± 1.2%, *n* = 4, Fig. 1a), and this response was not modified by inhibitors of prostanoids, NO and EDH production (Indomethacin, 94.1 ± 1.2%; Indomethacin + L-NAME, 88.7 ± 3.5%; Indomethacin + L-NAME + TEA, 89.6 ± 3.4%), suggesting the endothelium is not stimulated during relaxation to levamisole (Fig. 1a).

In order to evaluate if ARs were involved in this relaxing response, levamisole-induced relaxation was studied before and after treatment with AR blockers. Addition of the selective α_1_-AR blocker prazosin (10^−6^ M) completely prevented maximum relaxation to levamisole, while yohimbine (10^−6^ M), a selective α_2_-AR blocker, shifted to the right the concentration–response curve to levamisole, diminishing pEC_50_ (from 3.89 ± 0.05 to 3.22 ± 0.02, control vs. yohimbine *P* < 0.01, Fig. 1b). Propranolol, a non-selective β-AR blocker, did not attenuate relaxation to levamisole. These data suggest that levamisole-induced relaxation is mediated by α-AR blockage.

### α_1_-AR Stimulation is Inhibited by Levamisole

**Fig. 2:**
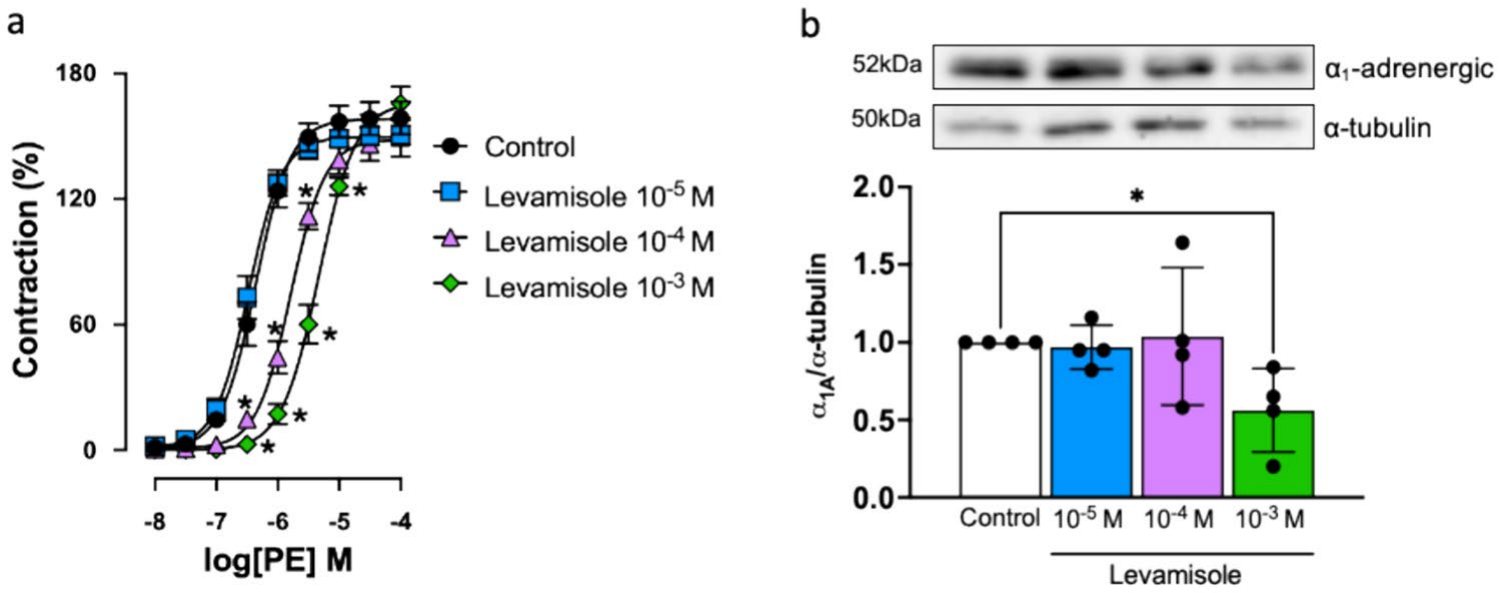
Levamisole impairs the activation of α_1_-AR. **a** Contraction–response curves to cumulative PE (10^−8^ to 10^−4^ M), an α_1_-AR agonist, in the absence (control) and presence of levamisole (10^−5^ to 10^−3^ M). Data are means ± SEM (*n* = 8 to 11 arteries from different rabbits). **b** Representative Western blot for α_1_-AR proteins in the absence (control) and presence of levamisole (Lev, 10^−5^ to 10^−3^ M); α-tubulin was used as internal control. Data are means ± SEM (*n* = 4 arteries from different rabbits); *P < 0.05, **P < 0.01 vs. control

Contraction to PE (10^−9^ to 10^−4^ M) was studied in the absence and presence of levamisole to assess its effect during α_1_-AR activation in renal arteries. PE caused concentration-dependent contraction, which was right-shifted in the presence of levamisole (10^−4^ to 10^−3^ M, Fig. 2a; Table 1), confirming the α_1_-AR antagonistic effects of levamisole. Furthermore, consistent with this reduced response to PE, α_1_-AR expression was down-regulated in the presence of levamisole (10^−3^ M, Fig. 2b).

**Table 1:** pEC_50_ values and maximal responses (*E*_max_) to phenylephrine in renal arteries in the different experimental conditions

Phenylephrine	*n*	pEC_50_	*E*_max_
Control	11	6.37 ± 0.2	157.1 ± 8
Levamisole 10^−5^ M	10	6.35 ± 0.3	150.5 ± 5
Levamisole 10^−4^ M	8	5.92 ± 0.3**	150.3 ± 11
Levamisole 10^−3^ M	8	5.37 ± 0.2****	165.7 ± 8

Data are shown as mean ± SEM. *E*_max_ are expressed as a percentage of response to 60 mM KCl

*n* Number of rabbits, *pEC*_*50*_ − log *M* of substance causing 50% of the maximal contraction

**P < 0.01 and ****P < 0.0001 vs. control

### Levamisole Modulates EFS-Induced Contraction

EFS was performed on renal arteries before and after the addition of levamisole. Under control conditions, EFS caused a contractile response which was attenuated by the addition of tetrodotoxin (10^−6^ M) to block voltage-gated Na^+^ channels, guanethidine (10^−6^ M) to block transmission in post-ganglionic adrenergic nerves and prazosin (10^−6^ M) to block α_1_-AR (data not shown), indicating a neurogenic contractile response causing the release of NA from perivascular nerves which activates α_1_-AR, and not a direct stimulus on vascular smooth muscle.

**Fig. 3:**
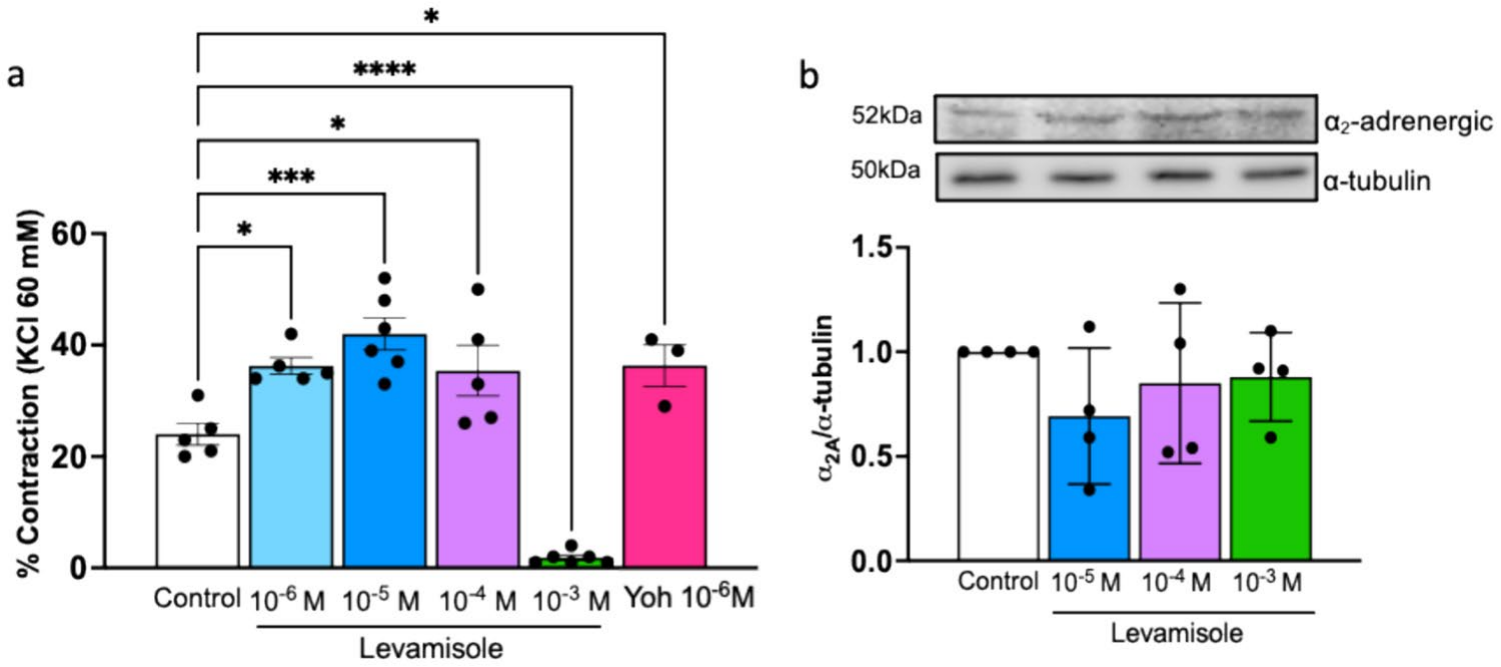
Levamisole enhances EFS-induced contraction but does not change α_2_-AR protein expression in rabbit renal arteries. **a** Bar graph summarizing EFS-induced contraction in the absence (control) and presence of levamisole (Lev, 10^−6^ to 10^−3^ M) and yohimbine (Yoh, 10^−6^ M). Data are means ± SEM (*n* = 3 to 6 arteries from different rabbits). **b** Representative Western blot for α_2_-AR proteins in the absence (control) and presence of levamisole (Lev, 10^−5^ to 10^−3^ M); α-tubulin was used as internal control. Data are means ± SEM (*n* = 4 arteries from different rabbits); *P < 0.05, **P < 0.01, ***P < 0.001 and ****P < 0.0001 vs. control

EFS-induced contraction was further increased by the addition of cumulative levamisole (Control, 24 ± 1.94%; levamisole 10^−6^ M, 36.2 ± 1.9%; levamisole 10^−5^ M, 42 ± 2.8%; levamisole 10^−4^ M, 35.4 ± 4.5%, Fig. 3a). This increased contractile response may be attributed to a blockade of α_2_-AR, as it mirrored the effect induced by the selective α_2_-AR blocker yohimbine (Fig. 3a). Nevertheless, levamisole did not decrease α_2_-AR protein expression in renal arteries (Fig. 3b). Moreover, the highest concentration of levamisole completely blocked EFS-induced contraction (levamisole 10^−3^ M, 1.8 ± 0.4%, Fig. 3a), which is in line with its antagonistic effect on α_1_-AR. These data suggest that levamisole acts as a presynaptic α_2_-AR blocker, increasing the contractile response to adrenergic stimulation while, at high concentrations, it blocks the postsynaptic α_1_-AR, thus decreasing the vascular adrenergic response.

### Levamisole Induces Endothelial Dysfunction by Reducing NO Bioavailability

**Fig. 4:**
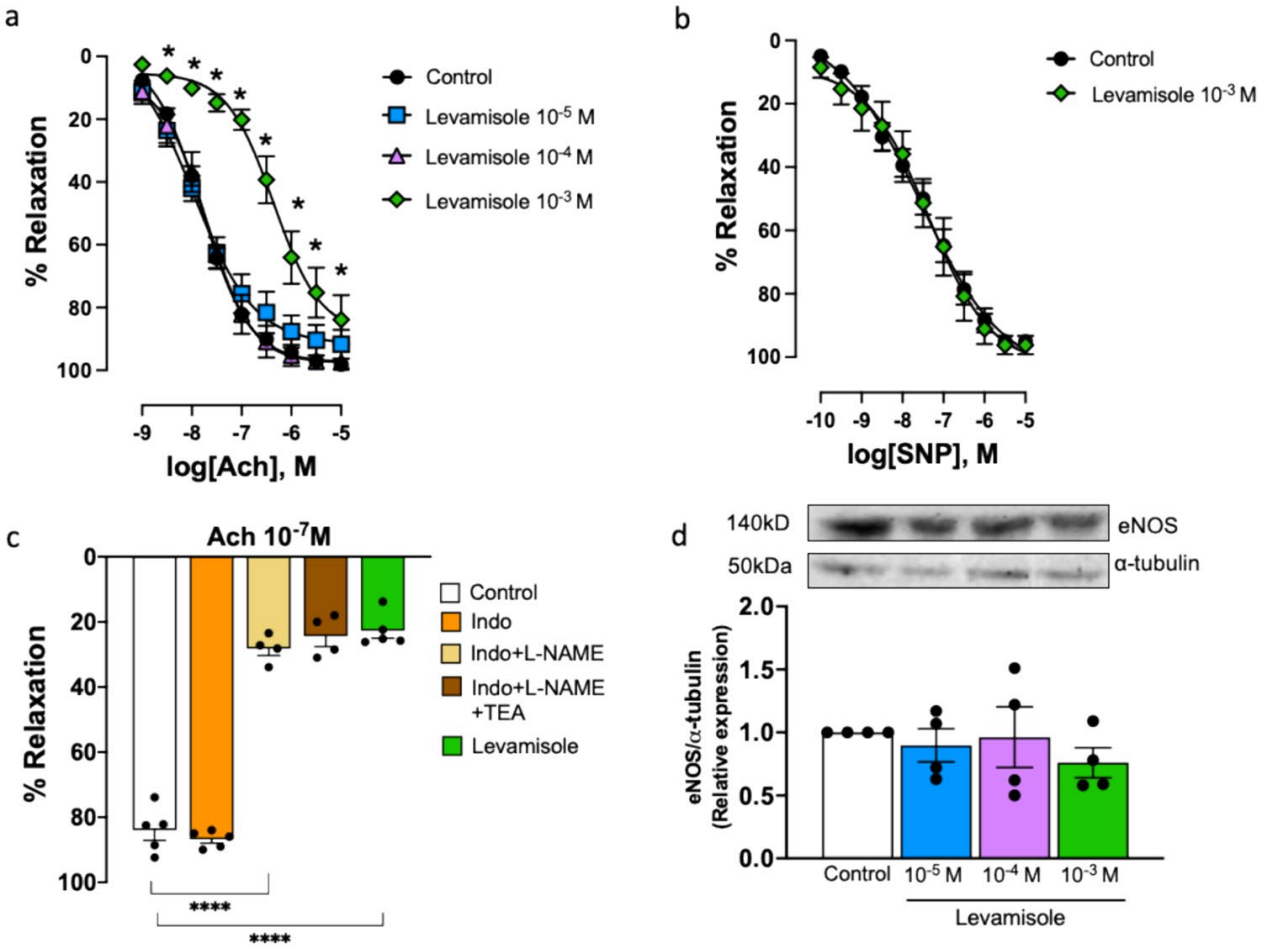
Levamisole impairs endothelium-dependent relaxation but does not change eNOS protein expression in rabbit renal arteries. **a** Relaxation–response curves to cumulative acetylcholine (ACh, 10^−9^ to 10^−5^ M), a M3 muscarinic receptor agonist that activates endothelial NO production, in the absence (control) and presence of levamisole (10^−5^ to 10^−3^ M). Data are means ± SEM (*n* = 5 to 11 arteries from different rabbits). **b** Relaxation–response curves to cumulative sodium nitroprusside (SNP, 10^−10^ to 10^−5^ M), a nitric oxide donor independent of endothelial NO synthesis, in the absence (control) and presence of levamisole (10^−3^ M). Data are means ± SEM (*n* = 5 to 11 arteries from different rabbits). **c** Bar graph summarizing relaxation to ACh (10^−7^ M) in the absence (control) and presence of indomethacin (Indo, 10^−5^ M), a non-selective COX inhibitor, as well as consecutive additions of L-NAME (10^−4^ M), to block nitric oxide synthase and tetraethylammonium (TEA, 10^−3^ M), a calcium-activated K^+^ channels blocker, and levamisole (10^−3^ M). Data are means ± SEM (*n* = 4 to 5 arteries from different rabbits). **d** Representative Western blot for eNOS proteins in the absence (control) and presence of levamisole (Lev, 10^−5^ to 10^−3^ M); α-tubulin was used as internal control. Data are means ± SEM (*n* = 4 arteries from different rabbits). *P < 0.05 and ****P < 0.0001 vs. control

As expected, levamisole impaired endothelium-dependent relaxation induced by ACh (10^−9^ to 10^−4^ M) in renal arteries (Fig. 4a; Table 2), but it did not modify endothelium-independent relaxation to the NO donor sodium nitroprusside (SNP, 10^−10^ to 10^−5^ M, Fig. 4b).

**Table 2:** pEC_50_ values and maximal responses (*E*_max_) to acetylcholine in renal arteries in the different experimental conditions

Acetylcholine	n	pEC_50_	*E*_max_
Control	11	7.84 ± 0.1	98.1 ± 1
Levamisole 10^−5^ M	5	7.93 ± 0.2	91.6 ± 4
Levamisole 10^−4^ M	5	7.70 ± 0.2	97.4 ± 2
Levamisole 10^−3^ M	6	6.48 ± 0.2****	83.9 ± 8

Data are shown as mean ± SEM. *E*_max_ is expressed as a percentage of response to 60 mM KCl

*n* Number of rabbits, *pEC*_*50*_ − log *M* of substance causing 50% of the maximal contraction

****P < 0.0001 vs control

ACh-induced relaxation is mostly dependent on NO in rabbit renal arteries, as was proved by a set of experiments where ACh (10^−7^ M), which stimulated sub-maximum relaxation, was studied before and after treatment with indomethacin, L-NAME and TEA (Fig. 4c). On this basis, the detrimental effect of levamisole could be due to a reduced NO bioavailability since impaired relaxation to ACh induced by levamisole evokes the same level of relaxation as the addition of NO blocker L-NAME (Fig. 4c). Nevertheless, intact renal arteries incubated with levamisole showed no evidence of a significant reduction in eNOS protein expression (Fig. 4d).

### Vascular Oxidative Stress Underlines Loss of Endothelial NO Induced by Levamisole

**Fig. 5:**
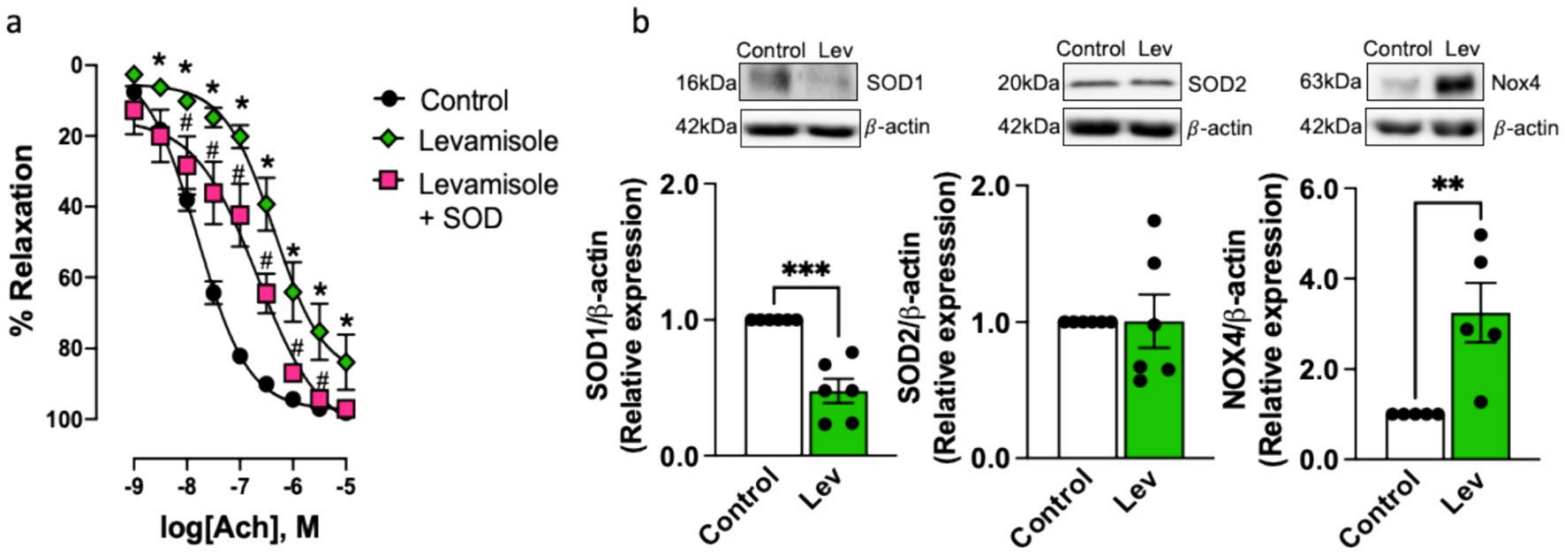
Oxidative stress underlies impaired endothelium-dependent relaxation by levamisole in rabbit renal arteries. **a** Relaxation–response curves to cumulative acetylcholine (ACh, 10^−9^ to 10^−5^ M), a M3 muscarinic receptor agonist that activates endothelial NO production, in the absence (control) and presence of levamisole (10^−3^ M) alone and levamisole plus SOD (200 U/ml). Data are means ± SEM (*n* = 5 to 11 arteries from different rabbits). **b** Representative Western blot for SOD1, SOD2 and Nox4 proteins in the absence (control) and presence of levamisole (Lev, 10^−3^ M); β-actin was used as internal control. Data are means ± SEM (*n* = 5 to 6 arteries from different rabbits). *P < 0.05 and ***P < 0.001 vs. control; ^#^*P* < 0.05 vs. levamisole

To demonstrate the role of vascular oxidative stress in impaired endothelium-dependent relaxation in the presence of levamisole, we treated renal arteries with SOD (200 U/ml) and levamisole (10^−3^ M). As shown in Fig. 5a, SOD partially prevented impairment of ACh-induced relaxation by levamisole, diminishing pEC_50_ (from 7.84 ± 0.1 to 6.60 ± 0.29, Levamisole vs. Levamisole plus SOD *P* < 0.01). This was consistent with reduced SOD1 but not SOD2 protein expression (Fig. 5b). Furthermore, Nox4, a main source of ROS in kidney tissue [30], was upregulated in renal arteries incubated with levamisole (Fig. 5b). Taken together, these results demonstrate that impaired antioxidant capacity and stimulation of Nox4 underlie loss of NO induced by levamisole.

## Discussion

The main finding of the present study is that levamisole impairs or enhances vascular tone in rabbit renal arteries, likely through inhibition of α_1_ or α_2_-AR, respectively, and causes endothelial dysfunction. Moreover, levamisole causes a loss of endothelial NO-dependent relaxation without a reduction in eNOS protein expression.

To our knowledge, this is the first study to demonstrate that levamisole induces endothelial dysfunction in the renal artery. Although this effect has been previously reported by our group in rabbit aorta [31] and by others in mouse aorta [32], it is well known that the mechanism of vascular adaptation varies from one vascular bed to another [33]. Therefore, the vascular response to a stimulus may be heterogeneous between different vascular beds.

Our previous study [31] demonstrated that levamisole induces relaxation in aorta rings contracted with NA but not in those contracted with ET-1, U46619 or high-K^+^. These results suggest that levamisole interferes with the selective mechanisms of vascular adrenergic contraction, either by blocking intracellular signal transduction pathways involved in vascular contraction or even by antagonizing AR [34]. The latter mechanism was directly tested in the present study by using prazosin and yohimbine, an α_1_ and α_2_-AR blockers. Our observation that prazosin completely blocked levamisole-induced relaxation, while yohimbine partially prevented this response, argues for an involvement of α-AR in the vascular action of levamisole. Furthermore, an additional characteristic of the levamisole-induced relaxation is its independence from endothelium-derived vasodilators, such as prostanoids, NO or EDH, as the relaxing effect of levamisole was preserved in renal arteries previously incubated with indomethacin, L-NAME and TEA. Because PE is a selective agonist of α_1_-AR expressed in smooth muscle that causes contraction and levamisole right-shifted this response and down-regulated α_1_-AR expression, it is reasonable to suggest that in smooth muscle, levamisole acts as an α_1_-AR blocker. These observations were consistent with the previous finding that levamisole reduced renal constriction to exogenous NA, a non-selective α_1_-agonist, in isolated perfused rat kidneys [35].

The renal arteries are extensively innervated by sympathetic nerves, which play an important role in the regulation of vascular tone [36]. NA is the main neurotransmitter released by sympathetic nerve endings. Its release is controlled by pre-junctional α_2_-AR, which act as autoreceptors that inhibit NA release [36]. Sympathetic overactivity is frequently associated with the development of acute kidney injury [37], which causes a reduction of renal blood flow. Therefore, an important question is whether the ability of levamisole to attenuate the adrenergic response induced by post-junctional α_2_-AR could also affect the contribution of α_2_-AR on pre-junctional regulation. We showed that levamisole enhanced EFS-induced contraction, similar to observations made with yohimbine, a selective α_2_-AR blocker. These data are consistent with what occurs in other vascular beds, such as the rabbit aorta [31]. However, the highest concentration of levamisole abolished this response. This finding may be related to a threshold point for post-junctional α_1_-AR blockade induced by levamisole. Interestingly, in contrast to our previous study [31], a non-significant reduction in the expression of α_2_-AR was observed, further suggesting that intracellular signalling involved in the sympathetic response to levamisole in rabbit renal arteries may also be influenced by other mechanisms, such as the NA reuptake blockade proposed by Hofmaier et al. (13). In this regard, although we did not perform experiments comparing whether levamisole mimics the effect of NA reuptake inhibitors, it is worth noting that the vascular effects of levamisole observed in our EFS experiments could be in line with this idea. Therefore, levamisole could increase the amount of NA available and, consequently, its effect.

On the other hand, we observed significant differences between the changes produced by levamisole on ACh-mediated relaxation that could be related to a loss of NO, since we demonstrated that NO synthesis is crucial for endothelium-dependent relaxation in renal arteries. However, investigations into the loss of NO induced by levamisole are scarce. A prior study reported that levamisole reduces eNOS protein expression in HUVECs [24]. Here, we observed no significant difference in renal arteries in terms of eNOS expression in the presence of levamisole, suggesting that the reduced response to ACh in the presence of levamisole could be due to a decrease in NO bioavailability. Indeed, NO produced by eNOS can be scavenged by excess reactive oxygen species, ROS [38]. Therefore, NO bioavailability depends on the balance between ROS in the vascular wall and NO production [39]. In this case, we observed a significant difference in the response to ACh between control renal arteries and those incubated with the highest concentration of levamisole in the presence of SOD, supporting that NO is partly quenched by levamisole-induced excess ROS. However, the increase in ROS could simply reflect a decrease in antioxidant capacity, which was ruled out as protein expression of SOD1 and Nox4 is likewise altered in renal arteries incubated with levamisole. Therefore, although it appears that NO production is altered due to levamisole, we cannot confirm this since we did not measure eNOS activity or nitrate and nitrite levels in the vascular ring, but our experiments indicate that levamisole increases vascular oxidative stress, and this could lead to the loss of NO bioavailability.

Taken together, our results provide evidence that levamisole, depending on its concentration, modulates renovascular tone by acting as a non-selective α-AR blocker in rabbits. In addition, NO bioavailability is reduced in the presence of levamisole likely due to increased local oxidative stress, leading to endothelial dysfunction. Finally, it is important to note that the renal artery does not regulate renal blood flow per se, and this could implicate a limitation of this study. However, the renal artery is the major conduit of blood flow to the kidney and is highly innervated. Changes in its diameter and tone will therefore influence overall blood flow into the kidney, impacting the renal microvasculature. Furthermore, while acknowledging that the study of vascular responses is more readily conducted in the main renal artery compared to resistance vessels, that understanding the impact of levamisole on renal artery functional responses will guide subsequent experiments aimed at understanding how this drug, through adrenergic receptor modulation, both pre and postsynaptic, and endothelial function, could impact intrarenal perfusion and vascular resistance and modulate responses to vasoactive stimuli within the kidney, given they are part of the same arterial tree. Therefore, we can conclude that levamisole would interfere with the response to vasoactive stimuli in the renal arterial tree and worsen the deleterious consequences of cocaine use.

## Data Availability

The data that support the findings of this study are available from the corresponding author upon reasonable request.
